# Genetic benefits of genomic selection breeding programmes considering foreign sire contributions

**DOI:** 10.1186/s12711-019-0483-5

**Published:** 2019-07-16

**Authors:** Daragh Matthews, John F. Kearney, Andrew R. Cromie, Fiona S. Hely, Peter R. Amer

**Affiliations:** 1 Irish Cattle Breeding Federation, Bandon, Co. Cork, Ireland; 2 AbacusBio Limited, PO Box 5585, Dunedin, 9058 New Zealand

## Abstract

**Background:**

In modern dairy breeding programmes, high contributions from foreign sires are nearly always present. Genotyping, and therefore genomic selection (GS), concern only a subpopulation of the breeding programme’s wider dairy population. These features of a breeding programme contribute in different ways to the rate of genetic gain for the wider industry.

**Methods:**

A deterministic recursive gene flow model across subpopulations of animals in a dairy industry was created to predict the commercial performance of replacement heifers and future artificial insemination bulls. Various breeding strategies were assessed by varying the reliability of breeding values, the genetic contributions from subpopulations, and the genetic trend and merit of the foreign subpopulation.

**Results:**

A higher response in the true breeding goal measured in standard deviations (SD) of true merit (*G*) after 20 years of selection can be achieved when genetic contributions shift towards higher merit alternatives compared to keeping them fixed. A foreign annual genetic trend of 0.08 SD of the breeding goal, while the domestic genetic trend is 0.10 SD, results in the overall net present value of genetic gain increasing by 1.2, 2.3, and 3.4% after 20 years as the reliability of GS in the domestic population increased from 0.3 to 0.45, 0.60 and 0.75. With a foreign genetic trend of 0.10 SD, these increases are more modest; 0.9, 1.7, and 2.4%. Increasing the foreign genetic trend so that it is higher than the domestic trend erodes the benefits of increasing the reliability of domestic GS further.

**Conclusions:**

Having a foreign source of genetic material with a high rate of genetic progress contributes substantially to the benefits of domestic genetic progress while at the same time reducing the expected returns from investments to improve the accuracy of genomic prediction in the home country.

## Background

Subpopulations of dairy cattle exist within every country’s dairy industry. Herd owners within these subpopulations tend to place emphasis on different aspects of their farming enterprises and therefore make different breeding decisions [1]. In addition, subpopulations are made up of cows that differ in average genetic merit. All breeding decisions made in these subpopulations contribute to the aggregate industry rate of genetic gain.

Genetic improvement in production traits of dairy cattle will contribute obvious economic benefits and benefits to the environment [2–5]. In 2001, Meuwissen et al. [6] proposed the use of genomic selection (GS) to increase the rate of genetic gain in animals. In dairy cattle, GS leads to higher accuracies of predicted genetic merit for young animals, which in turn typically lead to shorter generation intervals through higher contributions from young genetically superior bulls and heifers, and to increased selection intensity since GS can be used to test larger groups of potentially elite animals than traditional progeny testing structures. These factors have been estimated to double the rate of genetic progress for economically important dairy traits [7, 8]. In American dairy cattle, GS has been demonstrated to increase genetic gain by ~ 50 to 100% for yield traits and from threefold to fourfold for lowly heritable traits [9]. Thus, GS has radically changed many dairy breeding programmes.

In a domestic dairy cattle industry, it is common that certain subpopulations of herds drive most of the genetic gain because, compared to other subpopulations, they place more emphasis on elite bulls that are identified through GS. For these subpopulations, more animals are genotyped, and more phenotypic data are collected, thus they can contain superior dams compared to the national average. These features often result in strong relationships with artificial insemination (AI) companies, which place a significant emphasis on breeding a new generation of high merit sires using calves generated within these herds. In contrast, the majority of herds within a dairy industry will be more commercially driven. Although genetic gain will affect the commercial performance of the herd, breeding decisions may be based more on management and/or short-term considerations of costs. For example, a commercially-driven farmer may not see the advantage in genotyping calves because of genotyping costs. They may make their selection decisions for replacement heifers on the basis of management factors such as date of birth, bodyweight at selection, etc., or they may only use daughter proven (DP) sires to breed replacements because of their higher reliabilities. Therefore, overall, these commercially-driven farmers make breeding decisions with less emphasis on genetic merit. Small-sized nucleus dairy cattle breeding programmes have been shown to be effective in developing countries where larger, national breeding programmes are more difficult to implement [10]. To date, how higher rates of genetic gain in smaller subpopulations affect the wider industry in developed countries has not been investigated.

Gene flow models are an effective methodology to determine the outcomes of breeding strategies. Santos et al. [11] investigated the genetic and economic benefits of performance recording and genotyping in different subpopulations of sheep in Australia using gene flow methodology based on the principles of multi-tiered breeding structures that were introduced many decades ago by Bichard [12]. Likewise, Hely et al. [13] used a similar gene flow model in an Irish scenario to predict the benefits of a maternally-focused breeding programme for the Irish beef industry. A significant advantage of using gene flow prediction models over the simpler four-pathway selection approach [14] is that they capture the time delays that are associated with the transition from one breeding programme state to another, rather than assuming an instantaneous shift in the rate of genetic gain achieved. This is particularly important when short generation interval breeding strategies such as GS are compared with long generation interval breeding strategies such as progeny testing, and when intermediate subpopulations exist between the main source of genetic gain and a large commercial population. While stochastic simulation modelling approaches can also model transitions, they can be cumbersome because of the long computing time required to evaluate each scenario.

In this work, we investigated a number of different options for selection within, and genetic contributions from, subpopulations within a generalised dairy industry to determine their impacts on the overall rate of industry genetic progress.

## Methods

### Overview of the model

A deterministic recursive model with multiple flows of predicted genetic merit across subpopulations of animals was created using Microsoft Excel, to predict the genetic merit of both breeding bulls and commercial cows in a country’s dairy industry. The unit used for genetic trend, and for levels of genetic merit and of selection differentials was standard deviations of the true breeding goal ($G$). The subpopulations created for this model are described in Table 1 and named active (*AC*), passive (*PV*) corresponding to the domestic cow population, and foreign (*FOR*) where only sires are relevant. Initially, the model was parameterised with mean values of $G$ for sires and dams, age distributions and sire usage statistics within subpopulations. Flows of genetic merit from the *AC* and *FOR* subpopulations to the *PV* subpopulation via sires sourced from each one were built into the predictive model. Starting mean values and selection differentials for each sire type were set in each subpopulation such that a rate of genetic gain of 0.10 $G$ was achieved by the base model scenario. This rate of genetic gain was chosen based on the observation that annual genetic trends in dairy industries represent typically 10 to 15% of the genetic standard deviation of the aggregate merit. The impacts of various changes in breeding strategy were assessed by how much these changes contribute to the total benefits within the entire domestic dairy industry after 20 years. Then, future industry selection strategies were evaluated by manipulating three features of the model: (1) change in the reliability of breeding values due to improved genomic prediction; (2) change in the flow of genetic contributions such that sires from subpopulations with higher genetic merit have a larger genetic impact on the industry; and (3) increase or decrease in the genetic trend and genetic merit of the *FOR* subpopulation at the start of any change in breeding policy.

**Table 1:** Subpopulations of dairy cattle included in the model

Full name	Abbreviation	Description
Active	*AC*	Herds that use high levels of GS and have the potential to breed future AI bulls
Passive	*PV*	Commercial herds that do not have interest or potential to breed future AI bulls
Foreign	*FOR*	Foreign born sires

### Inputs

Cows from the *AC* subpopulation had a starting mean value of 0 $G$. All other starting mean values were set relative to the *AC* cows and are in Table 2. To calculate the industry average merit and the benefits associated with this, the *AC* subpopulation comprised 10% of the total industry population. The remaining 90% corresponded to the *PV* subpopulation. The *FOR* subpopulation was not included as a proportion of the total industry cow population since it contributes only through sire matings.

**Table 2:** Parameter description, units and values for key inputs driving the recursive model of estimated benefits

Parameter description	Units	Value
Standard deviations of the true breeding goal ($G$)	Standard deviations	1
Base genetic trend	Standard deviations	0.100
Base genetic trend in *FOR*	Standard deviations	0.105
Starting genetic merit of *PV* cows	Standard deviations	− 0.65
Starting genetic merit of *PV* SB sires	Standard deviations	− 0.59
Percentage of cows from *PV* herds sired by SB	Percentage	5
Base *AC* GS sire reliability	Reliability	0.45
Base *AC* DP sire reliability	Reliability	0.80
Base *FOR* sire reliability	Reliability	0.70

*FOR* foreign subpopulation, *PV* passive subpopulation, *AC* active subpopulation, *SB* stockbull, *GS* genomic selection, *DP* daughter proven

The starting mean value for *AC* sires was calculated as a deviation from the mean value for *AC* cows (zero in the equation below) due to a time-lag difference in genetic trend and to the selection differential achieved in cows, as follows:

$$G_{W,Y = 0}^{Sires} = 0 + \left( {\overline{X}_{AC}^{Cows} } \right. - \left. {\overline{X}_{W}^{Sires} } \right)*$$

1 $$\Delta^{Dom} + \mathop \sum \limits_{i = 1}^{10} \left[ {SDcows_{i}^{AC} * \gamma_{i}^{AC,cows} } \right],$$

where $\overline{X}$ is the mean age of a group of animals of a specific sex (sires or cows) within a subpopulation, $W$ is the source of sires (GS or DP in this case), $\Delta^{Dom}$ is the current rate of genetic gain across the entire domestic cow population and which is the same within *AC* and *PV* subpopulations, $SDcows_{i}^{AC}$ is the selection differential for cows in the *AC* subpopulation (see below) and $\gamma_{i}^{AC,cows}$ is the proportion of cows in the *AC* subpopulation that are of age $i$. In the base scenario, the starting mean value for *FOR* sires was made equal to domestic DP sires.

The genetic merit of AI calves born in the *AC* subpopulation in year 0 was calculated as:

2 $$\begin{aligned} G_{AC, Y = 0}^{Calves} &= G_{AC, Y = 0}^{Cows} + \left( {\overline{X}_{AC}^{Cows} } \right.* \left. {\Delta^{Dom} } \right)\\ &\quad - \mathop \sum \limits_{i = 1}^{10} \left[ {SDcows_{i}^{AC} * \gamma_{i}^{AC,cows} } \right]. \end{aligned}$$

In this way, the *AC* calves born in year 0 are superior over the genetic merit of cows born in the same year according to the current genetic trend occurring in the population. The selection differential from the cows, $SDcows_{i}^{AC}$, is removed as, at this stage, these are all calves born into the *AC* subpopulation and not just those selected to become replacement heifers. The genetic merit of selected AI calves born in the *AC* subpopulation in year 0 was calculated as:

3 $$G_{AC, Y = 0}^{SelectedCalves} = G_{AC, Y = 0}^{Calves} + \mathop \sum \limits_{W = 1}^{3} \frac{{\tau_{AC, W} * SDselectedcalves_{W} }}{2},$$

where $\tau_{AC,W}$ is the proportion of *AC* calves that were sired by an AI sire originating from three different sire sources denoted $W$. Sources of sires were *AC* genomically selected (GS), *AC* daughter proven (DP), and *FOR*. $SDselectedcalves_{W}$ is the selection differential for calves specific to each sire source of which only one half is transferred to calves. Methods used to calculate selection differentials are outlined below.

The starting genetic merit of *PV* AI and stockbull (SB) sired calves was calculated using the same algorithm as for future predicted merit values and is explained below.

Mean genetic values for years prior to implementation of new model scenarios at year $Y$ = 0 were computed assuming that the merit of all subpopulations was evolving at a genetic trend of 0.10 $G$ per year. Values of genetic merit of *PV* cows and SB sires in year 0 were set through trial and error such that a rate of genetic gain of 0.10 $G$ was achieved by the base model scenario. Resulting values are in Table 2 and the age profiles of the different animal types in the subpopulations are in Table 3.

**Table 3:** Proportion of calves born by sires and dams of different ages for animal groups

Age	*AC* GS sires	*AC* DP sires	*A*C dams	*PV* SB sires	*PV* dams
2	0.33	0	0.21	0.25	0.21
3	0.34	0	0.20	0.25	0.18
4	0.33	0	0.20	0.25	0.16
5	0	0.20	0.15	0.15	0.13
6	0	0.20	0.12	0.10	0.11
7	0	0.30	0.07	0	0.07
8	0	0.20	0.05	0	0.05
9	0	0.10	0	0	0.04
10	0	0	0	0	0.05

*AC* active subpopulation, *PV* passive subpopulation, *GS* genomic selection, *DP* daughter proven, *SB* stockbull

The mean reliabilities for dams at different ages in the subpopulations are in Table 4.

**Table 4:** Reliability of dam total index predictions at different ages

Dam age	*AC* dams	*PV* dams
2	0.35	0.30
3	0.38	0.33
4	0.40	0.35
5	0.41	0.36
6	0.45	0.40
7	0.45	0.40
8	0.45	0.40
9	0.45	0.40
10	0.45	0.40

*AC* active subpopulation, *PV* passive subpopulation

### Contributions of sires to subpopulations

In order to account for the lag between selection decisions and the flow of genetic contributions on the benefits from using bulls of each type, the proportion of calves by sires at different ages in each subpopulation was modelled. Each scenario was investigated by using fixed sire contributions (FSC) and responsive sire contributions (RSC). Fixed sire contributions remained unchanged throughout the timeframe of the model and were equal to 0.33, 0.34, and 0.33 for *AC* GS, *AC* DP and *FOR* sires in the base scenario, respectively.

Responsive sire contributions for *FOR* sires were calculated as:

4 $$\vartheta_{For}^{Sires} = \vartheta base_{For}^{Sires} + \frac{{\left( {G_{For}^{Sires} } \right. - \left. {G_{AC, DP}^{Sires} } \right)}}{2},$$

where $\vartheta$ is the proportion of total sire contributions from a sire type, and $G$ is the genetic merit of a particular group of animals (explained in detail below). All computations of $\vartheta$ were constrained to a minimum value of 0 and a maximum value of 1.

Initially, identical sire contributions for *AC* GS and DP sires were assumed and calculated as:

5 $$\vartheta_{W}^{Sires} = \frac{{\left( 1 \right. - \left. {\vartheta_{For}^{Sires} } \right)}}{2},$$

for sires from source $W$.

When RSC is used, the selection differentials for GS and DP sires are updated as the contributions of sires change, which is represented by the proportion of calves selected to become sires:

6 $$SelectedProp_{W}^{Sires} = \frac{{\vartheta_{W}^{Sires} }}{{\vartheta base_{W}^{Sires} }}*SelectedPropBase_{W}^{Sires} .$$

The reason for this is that, as the contributions from a sire type drops, the proportion of calves selected to become sires also drops, which leads to an increase in selection intensity. Selection proportions remain the same throughout the timeframe of the model for *FOR* calves and all sire types in the FSC scenarios.

### Recursive prediction of genetic merit in subpopulations and estimation of industry benefits

Future genetic trends in *AC* and *PV* subpopulations are estimated recursively from the genetic merit $G$ of the future dams. Table 5 describes the steps necessary to calculate predictions. Benefits per cow calving are then multiplied by the industry-wide numbers of cow calvings that are impacted, i.e. 1 million. Results from the model can be easily scaled according to cow population size by multiplying by the number of millions of cows of interest. The model accounts for the delays and lags for genetic selection decisions that occur at a high level in the breeding structure to cascade down to cows over time. Cumulative discounted benefits were calculated by considering the benefits after 10 and 20 years of selection. Because of the permanent and cumulative nature of genetic improvement, these benefits were augmented in each case by assuming that the genetic merit achieved at the end of the investment period (i.e. after 10 or 20 years) would be sustained for a further 5 years. The cumulative benefits were then converted into an annualised equivalent by calculating the annual flow of discounted benefits that would provide the equivalent return as the breeding programme over a 10- or 20-year period.

**Table 5:** Description of recursive method flow

Step	Action	References
1	Starting values for *AC* cows set at 0 $G$	
2	Starting values for *PV* cows and *PV* SB sires using lagged differentials	Table 2
3	Starting values for GS and DP *AC* sires calculated as the deviation from the mean value for *AC* cows due to a time lag difference in genetic trend, and the selection differential achieved in cows	Equation 1
4	Starting values of *FOR* sires made equal to *AC* DP sires (in the base scenario)	Table 2
5	Starting values for calves born in the *AC* subpopulation calculated as a time lag difference in genetic trend versus *AC* cows	Equation 2
6	Starting values for calves born in the *AC* subpopulation and selected to become future AI sires calculated as a time lag difference in genetic trend versus *AC* cows plus the selection differential achieved by cows	Equation 3
7	Historical trends are set for all subpopulations as increasing by 0.1 per year	Table 2
8	Select domestic sires from historical calves based on selection differentials and reliability	Equations 14 and 15
9	Update the genetic merit of foreign sires based on the foreign sire genetic trend	Equation 16
10	Evaluate the genetic merit of sires for each population and allocate the relative contributions	Values included in text for FSC. Equations 4 and 5 for RSC
11	For the RSC scenarios, update the selection differentials	Equation 6
12	Predict the genetic merit of born *AC* and *PV* calves from the merit of the corresponding sires and cows based on relative ages and sire contributions	Equations 11 and 13
13	Predict the genetic merit of *AC* and *PV* cow populations, and *PV* SB sires, based on lagged ages relative to calves	Equations 10 and 12
14	Cycle through steps 8 to 13 over successive years of the simulation	
15	Calculate the NPV of the industry benefits	Equations 7, 8 and 9

$G$ standard deviations of true merit, $AC$ active subpopulation, *PV* passive subpopulation, *SB* stockbull, *GS* genomic selection, *DP* daughter proven, *FOR* foreign subpopulation, *FSC* fixed sire contributions, *RSC* responsive sire contributions, *NPV* net present value

Cumulative benefit (expressed in $G$) from the national herd at the end of the investment period year ‘$nY$’ for each scenario of breeding scheme was calculated as:

7 $$CB_{Y} = \mathop \sum \limits_{Y = 1}^{nY} \frac{{AB_{Y} }}{{\left( {1 + r} \right)^{Y} }} + \mathop \sum \limits_{Y = nY + 1}^{nY + 5} \frac{{AB_{nY} }}{{\left( {1 + r} \right)^{Y} }},$$

where $r$ is the discount rate, taken as equal to 0.07, and $AB$ is the annual farm benefits (see Eq. 8 below) from the genetic improvement in year $Y$ that follows the start of the new breeding strategy, which is a function of the weighted (by cow type) average genetic merit in the national index in the base year ($G_{I, 0}$) and after $Y$ years of selection ($G_{I,Y}$), i.e.:

8 $$AB_{Y} = 2* \left( {G_{I, Y} - \left. {G_{I, 0} } \right)} \right..$$

A multiplication factor of 2 is included in Eq. 8 under the assumption that the breeding goal units are expressed as progeny differences, which reflect half of the total benefits when animals express the predicted genetic merit themselves.

Genetic response values of calves (measured in units of $G$) are computed recursively by modelling flows of genetic superiority within and among subpopulations of the industry dairy population. The weighted (by cow type) average industry genetic merit for $G$ is calculated by assuming that cows are made up of a proportion from the *AC* herds and the balance from the *PV* herds such that:

9 $$G_{I,Y} = G_{AC,Y}^{Cows} * \varphi_{AC} + G_{PV, Y}^{Cows} *\left( {1 - \varphi_{AC} } \right) ,$$

where $G_{AC, Y}^{Cows}$ and $G_{PV, Y}^{Cows}$ are the modelled predicted genetic merit for cows from *AC* herds in year $Y$ making up the proportion $\varphi_{AC}$ of the industry, and for *PV* herds, respectively.

The genetic merit for the breeding herd of cows in any subpopulation $X$ is calculated from the genetic merit of calves that were historically born in that subpopulation, either to SB or AI sires, as:

10 $$\begin{aligned} G_{X, Y}^{Cows} = \mathop \sum \limits_{i = 1}^{10} \left[ {\left( {G_{X, Y - i}^{SBcalves} * \rho^{X, Cows} + G_{X, Y - i}^{AIcalves} *\left( {1 - \rho^{X, Cows} } \right)} \right)} \right. \hfill \\ \left. {* \gamma_{i}^{X, Cows} + SDcows_{i}^{X} } \right], \hfill \\ \end{aligned}$$

where $\rho^{X,Cows}$ is the proportion of calves of subpopulation $X$, which are destined to become replacement cows that are sired by SB as opposed to being sired by AI, $\gamma_{i}^{X, Cows}$ is the proportion of the cows in a herd that are of age $i$ and $SDcows_{i}^{X}$ is the selection differential for cows that are selected to become replacements relative to the average merit of calves born in the same year as those selected to become replacements.

The genetic merit of calves born in the *PV* subpopulation, sired by SB and born in year $Y$ is calculated as:

11 $$G_{PV, Y}^{SBcalves} = \frac{{G_{PV, Y}^{Cows} + G_{PV, Y}^{SB} }}{2},$$

where the genetic merit of SB sires, $G_{PV, Y}^{SB}$ (Eq. 12), is in turn calculated from the weighted average genetic merit of calves weighted according to the age distribution for SB sires as:

12 $$G_{PV,Y}^{SB} = \mathop \sum \limits_{i = 1}^{10} \left[ {G_{PV,Y - i}^{AIcalves} * \gamma_{i}^{PV, Bulls} } \right],$$

where $\gamma_{i}^{PV, Bulls}$ is the proportion of the SB sires used in a herd that are of age $i$. While the previous two equations have interdependencies, the recursive and time lagged nature of the equations make the calculation tractable from a fixed historical trajectory of levels of genetic merit.

The genetic merit of AI calves in year $Y$ depends on which other populations have contributed sires to the pool that is used to mate cows in subpopulation $X$ and is calculated as:

13 $$G_{X, Y}^{AIcalves} = \frac{{G_{X, Y}^{Cows} + \mathop \sum \nolimits_{W = 1}^{3} \left[ {\tau_{X,W} * G_{W, Y}^{Sires} } \right]}}{2},$$

where $\tau$ is the proportion of calves in subpopulation $X$ that were sired by an AI sire originating from sire source $W$. Sources of sires were considered to be *AC* GS, *AC* DP, and *FOR*.

The genetic merit of sires from the *AC* subpopulation is calculated as:

14 $$G_{AC, Y}^{Sires} = \mathop \sum \limits_{i = 1}^{10} \left[ {\gamma_{i}^{AC, Sires} * G_{AC, Y - i}^{SelectedCalves} } \right],$$

where $\gamma_{i}^{AC, Sires}$ is the proportion of sires that originate from the *AC* subpopulation at each age $i$ when their calves are born ($\gamma_{i}^{AC, Sires}$ differs between *AC* GS and *AC* DP sires) and $G_{AC, Y}^{SelectedCalves}$ (Eq. 15) is calculated as:

15 $$G_{AC,Y}^{SelectedCalves} = \frac{{G_{AC, Y}^{Cows} + \mathop \sum \nolimits_{W = 1}^{3} \left[ {\tau_{X,W} *\left( {G_{W, Y}^{Sires} + SDselectedcalves_{W} } \right)} \right]}}{2}.$$

The genetic merit of sires that are sourced from foreign populations denoted $W = F$ is calculated as:

16 $$G_{W = F, Y}^{Sires} = G_{Y = 0}^{ForSires} + \left( {Y*\Delta^{For} } \right),$$

where $\Delta$ is the annual rate of improvement of $G$ of foreign sourced bulls.

### Selection differentials

Selection differentials for a class of selection candidates, $K$, were calculated as:

17 $$SD_{K} = i_{K} * r_{K} * f_{K} * G,$$

where $i$ is the intensity of selection, $r$ is the accuracy of selection, and $f$ is an adjustment for the reduction in standard deviation due to highly selected parents.

Accuracies were obtained by taking the square root of reliabilities modelled for the respective categories of selection candidates (listed in Table 2) while the adjustment to the selection differential to account for selection of parents was:

18 $$f_{K} = \frac{{\left( {G_{AC,Y = 0}^{GSsires} } \right. - \left. {\mathop \sum \nolimits_{i = 1}^{10} \left[ {\gamma_{i}^{AC, Sires} * G_{AC, Y - i}^{AIcalves} } \right]} \right)*2}}{{i_{K} * r_{K} * G}}.$$

### Scenarios

We investigated several scenarios that reflected various combinations of changes in breeding value reliability, sire usage proportions or improvements in foreign genetic trend and/or starting *FOR* sire genetic merit. Sire contribution methods (RSC and FSC) were compared to each other by adjusting two key variables. First, the impact of improvement in GS reliability was evaluated using reliability values for total economic merit of 0.3, 0.45, 0.6, and 0.75. Second, the impact of foreign genetic trend was evaluated using annual trend values of 0.08, 0.1, 0.12, and 0.14. For these scenarios, it was assumed that all domestic sire contributions were split evenly between GS and DP sires, since factors such as confidence, risk, and past habits drive farmer preferences between GS and DP sires, rather than the levels of genetic merit within the two groups of sires.

The impacts of increasing GS relative to DP sire contributions were evaluated for a subset of scenarios in which the foreign genetic trend was set to be equal to the domestic trend of 0.10. The range of GS reliability values (0.3, 0.45, 0.6, and 0.75) were tested at three proportional splits of domestic GS and DP sire contributions. The modelled breakdown between GS and DP sires considered were: 0.5 each; 0.5 increasing to 0.9 by 0.08 per year for GS at the expense of DP; and 0.8 GS sires and 0.2 DP sires from year 1.

A further set of scenarios was created to consider a closed dairy industry that was subsequently opened to imported foreign semen and/or sires, for the first time. For these scenarios, foreign genetic trends of 0.08, 0.1, and 0.12 were tested with the starting genetic merit of the *FOR* subpopulation being either higher than that of domestic sires at 0.5 or lower than that of domestic sires, but equal to that of *AC* dams at 0.

## Results

**Fig. 1:**
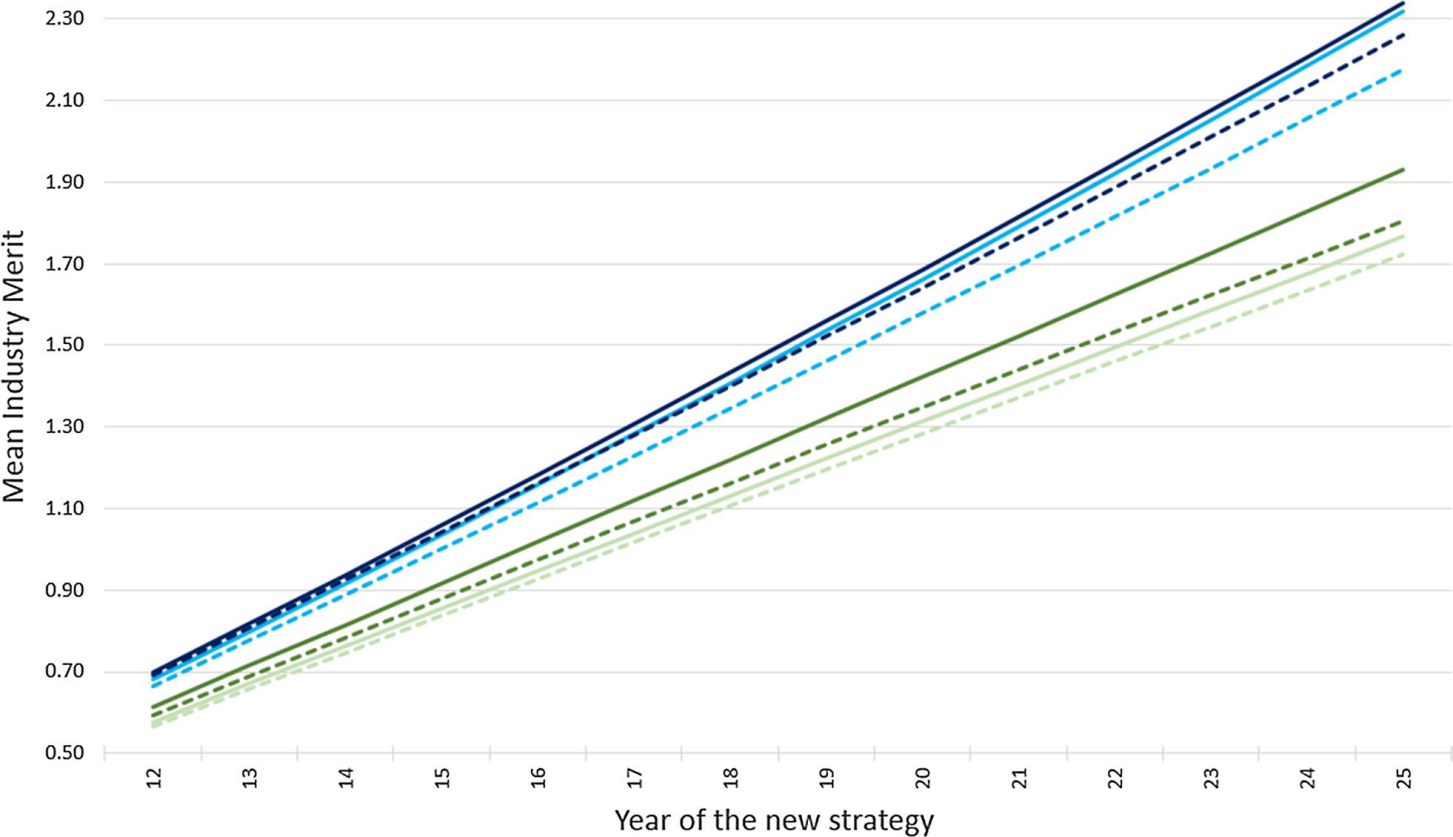
Mean industry genetic merit from years 12 to 25 of the new selection strategy for scenarios with high (blue, 0.14) or low (green, 0.08) foreign genetic trend, responsive (solid) or fixed (dashed) sire contributions, and high (dark, 0.75) or low (light, 0.30) genomic selection reliability

The mean genetic merit of commercial cows (*PV*) from years 12 to 25 of the new selection strategy for scenarios with a high (0.14) or low (0.08) foreign genetic trend, responsive or fixed sire contributions, and a high (0.75) or low (0.30) genomic selection reliability are presented in Fig. 1. Years 0 to 11 are not represented in Fig. 1 since differences in cow genetic merit up to year 12 were minimal. This slow impact of the new selection strategies is caused by gene flow lags with benefits due to a change in the breeding program taking several years to reach a large proportion of the commercial cow population. As shown in Fig. 1, the foreign genetic trend has a larger impact on the mean industry genetic merit than GS reliability. The effect of responsive sire contributions depends on the interaction between GS reliability and foreign genetic trend. At a low foreign genetic trend, RSC allows the use of more GS sires as GS reliability improves. At a high foreign genetic trend, RSC allows the use of more foreign sires in the situation of low GS reliability.

**Fig. 2:**
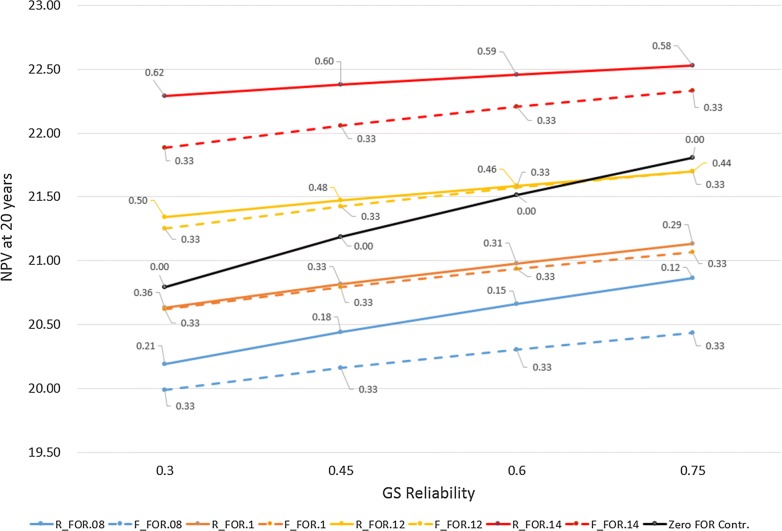
Predicted cumulative net present value at 20 years in units of standard deviation of the breeding goal expressed per cow at different foreign genetic trends, GS reliabilities and sire contribution types. R for responsive sire contributions; F for fixed sire contributions; FOR for foreign genetic trend. The levels of foreign sire contributions after 20 years are annotated onto the corresponding data point

The cumulative net present values after 20 years of modelling for each new scenario comparing RSC and FSC are in Fig. 2. In all scenarios, except one scenario for which results are equal, RSC delivers a higher genetic response (measured in standard deviations of the breeding goal) at 20 years compared to FSC when scenarios have the same GS reliability and foreign genetic trend. For example, using a foreign genetic trend of 0.08 with the domestic genetic trend being equal to 0.10, the NPV of genetic gain after 20 years of selection is increased by 1.2, 2.3 and 3.4% when the GS reliability is increased from 0.3 in steps of 0.15 to 0.45, 0.6 and 0.75, respectively, when sire contributions are calculated by RSC (Fig. 2). For the same scenarios, but using FSC, the increases were 0.95, 1.6, and 2.2% after 20 years with the same increases in GS reliability. With a higher foreign genetic trend of 0.1, the impacts on the cumulative NPV of genetic gain at year 20 of increasing GS reliability are more modest (0.9, 1.7, and 2.4% with RSC, and 0.8, 1.6, and 2.2% with FSC). At a foreign genetic trend of 0.12, the scenarios return results of 0.6, 1.2, and 1.7% increase per cow for RSC after 20 years. Finally, scenarios using a foreign genetic trend that is substantially larger than the domestic trend, 0.14 for foreign versus 0.10 for domestic, have NPV percentage increases of 0.4, 0.8, and 1.1% per cow after 20 years for RSC. Increasing the foreign genetic trend so that it is higher than the domestic trend erodes the benefits of increasing domestic GS reliability further, and consequently the magnitude of the difference between RSC and FSC is also reduced (Fig. 2).

For a situation that modelled *FOR* sires as having no contribution and instead all contributions being split equally between domestic GS and domestic DP sires, the NPV of genetic gains increased by 1.9, 3.5, and 4.9% with increasing GS accuracy. Increasing GS reliability has the largest impact on cumulative NPV when the foreign sire contributions are fixed at zero.

Figure 2 also shows the effect of increasing GS reliability for different foreign genetic trends, and how the foreign sire contributions at 20 years changed under the RSC scenarios. At a high foreign genetic trend, higher GS reliability reduces moderately the relative impacts of foreign sires. At a low foreign genetic trend, high GS reliability reduces the foreign sire contributions after 20 years to a greater extent.

**Fig. 3:**
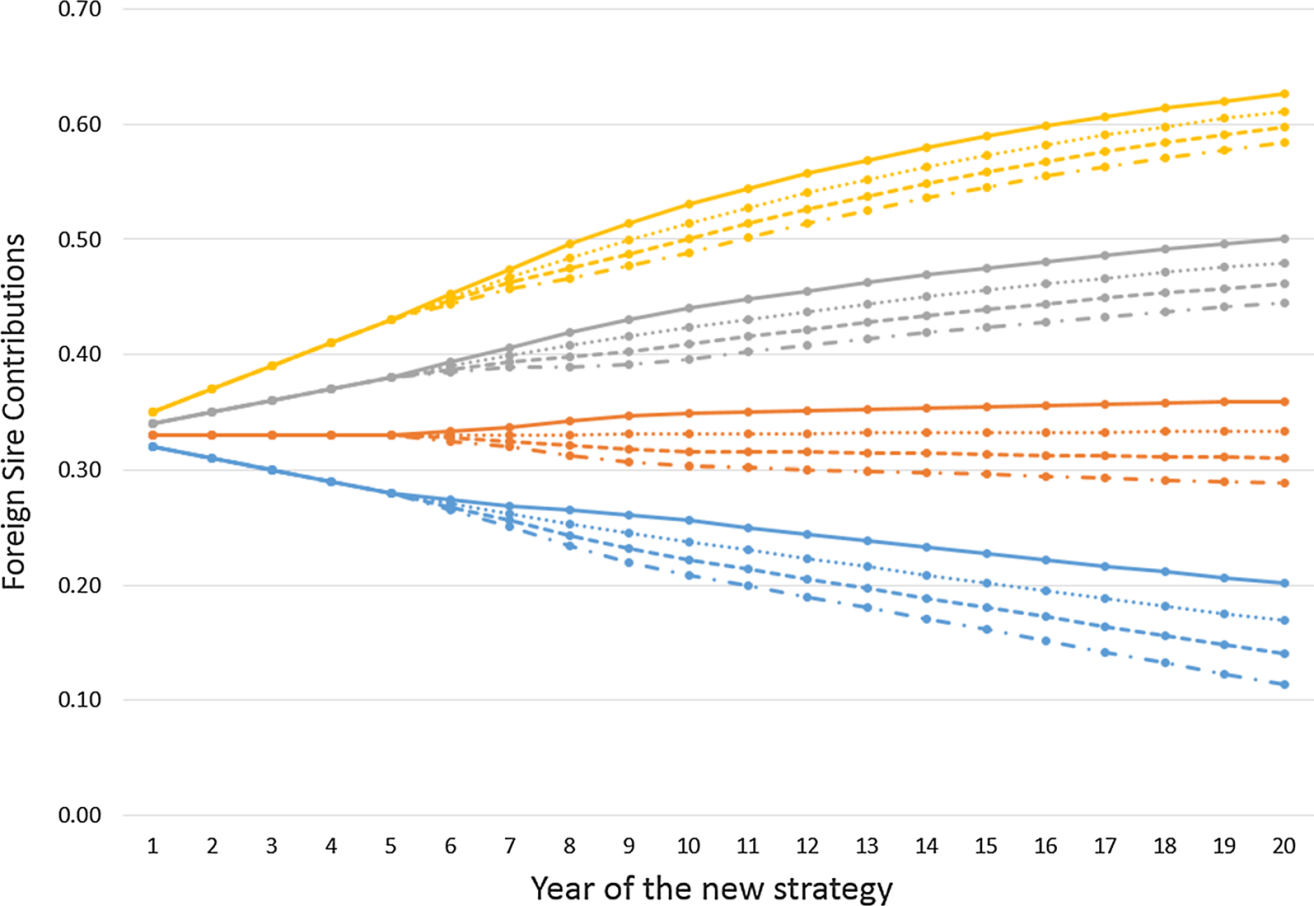
Foreign sire contributions over time for different scenarios using responsive sire contributions. Foreign genetic trends used in included scenarios are equal to 0.14 (gold), 0.12 (grey), 0.10 (orange), and 0.08 (blue). Genomic selection reliability used in included scenarios are 0.3 (solid), 0.45 (round dot), 0.6 (short dash), and 0.75 (dash dot)

The time trajectory of foreign sire contributions for the different scenarios are shown in Fig. 3. The foreign genetic trend has a bigger impact on these trajectories than does the GS reliability, which is partly due to the delay before the higher GS reliability increases the merit of candidate bulls of breeding age.

Table 6 shows the impacts of increasing GS sire contributions, at the expense of DP sire contributions, on the benefits that arise from improving GS reliability. The trend is similar for all scenarios, with NPV increasing as the modelled GS reliability increased but NPV increasing at a steeper rate when GS sires have higher contributions. The percentage differences per cow relative to the scenario with the lowest modelled GS reliability were 0.9, 1.7, and 2.4% for domestic sire contributions of 0.5 each for GS and DP sires; 1.5, 2.9, and 4.2% for sire domestic contributions of 0.5 increasing to 0.9 for GS sires at the expense of DP sires; and 1.5, 2.9, and 4.3% for sire domestic contributions of 0.8 for GS versus 0.2 for DP sires.

**Table 6:** Predicted NPV for scenarios with alternative GS sire contributions at different levels of GS reliability

GS reliability	0.5	0.5–0.9	0.8
0.3	20.63	21.25	21.32
0.45	20.82	21.58	21.65
0.6	20.98	21.87	21.95
0.75	21.13	22.15	22.23

Results reported in units of standard deviation of the true breeding goal per cow at 20 years. Modelled levels of the GS sire use are static 0.5, increasing from 0.5 to 0.9, and static 0.8. NPV: net present value; GS: genomic selection

The results of the scenario for an industry that imports foreign genetics for the first time are in Table 7. Scenarios using a high starting genetic merit for *FOR* sires and a low foreign trend have an increased cumulative NPV after 20 years compared to a base scenario that has no *FOR* sire contributions. The increased NPV relative to a base scenario with no *FOR* sire contributions increased further, as the modelled foreign trend increased to levels that were similar to that of the domestic trend (0.1) or higher (0.12), while starting foreign sire merit was high. When starting foreign sire merit and the foreign genetic trend are both low, cumulative NPV is reduced compared to a base scenario that has no *FOR* sires. However, with a low starting *FOR* sire merit, cumulative NPV increases when the modelled foreign genetic trend is the same as, or higher, than the domestic trend. This is somewhat surprising, especially in the scenario in which the foreign genetic trend is equal to that of the domestic sires but the foreign sire starting merit is low. However, here the low starting merit of foreign sires is still higher than that of domestic DP sires and therefore gains can be achieved by using these sires.

**Table 7:** Predicted cumulative NPV relative to a base scenario with zero foreign sire contributions

Foreign genetic trend	RSC	FSC	RSC	FSC
	High (0.5)		Low (0.2)	
Low (0.08)	2.21	1.82	− 0.02	− 0.11
Equal (0.10)	3.11	2.46	0.54	0.52
High (0.12)	4.22	3.09	1.37	1.15

Results reported in units of standard deviation of the true breeding goal per cow at 20 years for scenarios modelling the impact of introducing foreign sires at high and low starting sire merit with low, equal, and high foreign genetic trends relative to the domestic trend and sire contribution type

*NPV* net present value, *RSC* responsive sire contributions, *FSC* fixed sire contributions

## Discussion

In 2006, Schaeffer showed that genomic selection can double the rate of genetic gain at significantly reduced costs compared to a traditional progeny testing strategy [7]. Since then, GS has become a widely used tool in dairy cattle breeding programmes worldwide. However, in many cases, genotyping, and therefore GS, concern only a subpopulation of the breeding programme’s wider dairy population. This is due to the costs involved in genotyping, herd management factors, or perhaps farmer preference or knowledge of GS. Therefore, we have developed a model to investigate the effects on genetic gain of focusing GS on a subpopulation of dairy cattle within a breeding programme that is strongly influenced by the importation of sires and/or semen from other countries.

The standard approach of modelling genetic gain in cattle populations, which was introduced by Rendel and Robertson [14], is steady state, and therefore does not account for the long time lags before changes in selection strategy flow through the population. These delays are exacerbated when subpopulations exist within a larger population because of the additional time lags for benefits to flow from bull breeding herds to the larger commercial population (the *AC* and *PV* subpopulations here). In the scenarios that we investigated, the levels of genetic merit in the *PV* subpopulation typically lag the *AC* subpopulation by 7 years (results not shown). These modelling issues could also be addressed by using stochastic simulation [15, 16], but these can be complex to build and computationally intensive, especially if subpopulations are considered. Our model can be parameterised from industry data and used to test efficiently a large range of scenarios. There is further potential to expand the model by subdividing a country’s dairy industry into an even larger number of smaller subpopulations such as research herds, contract mating herds, etc., to investigate how the breeding decisions made in these subpopulations result in greater industry genetic gain.

The model presented here includes foreign sire contributions by taking the foreign genetic trend into account. Although other informative deterministic models have been created to look at GS strategies for dairy cattle populations [17], to our knowledge, none of these considers foreign sire contributions. Our results show the importance of accounting for the impact of these foreign sires in simulation studies, with our results showing substantial reductions in return on investments to increase GS reliability. This, along with the time delay for genetic gains to flow through to the *PV* cow population, closely mimics real world scenarios, i.e. in nearly all modern dairy breeding programmes, where very high contributions from foreign sires are present. Likewise, in our model, responsive sire contributions attempt to mimic real world scenarios. Sire contributions are a product of farmer breeding decisions and therefore are difficult to control at an industry level. Thus, our RSC approach is an attempt to quantify what might occur in practice as the domestic population shifts in merit relative to the foreign population. Another approach would have been to model truncation selection across the domestic and foreign sires available according to simulated overlapping distributions with means based on respective levels of genetic merit. This could be considered in a future study.

The value of increasing GS reliability when considered as cumulative NPV is surprisingly modest (0.9 to 3.4% depending on the parameters) when the magnitude of the increase (0.3–0.75) in reliability is considered. While the lags modelled are partly driving this surprisingly low return, the impact of foreign semen was found to be a major driver. The financial commitment to bring about an increase in GS reliability is substantial. Return on investment may not be realised if foreign genetic trend is high relative to the domestic trend and foreign semen is readily available. Having a foreign source of genetic material with a high rate of genetic progress contributes substantially to the domestic rate of genetic progress.

Our results show that investments to improve GS reliability will yield greater returns when the foreign genetic trend is lower than the domestic trend, which is most likely due to GxE interactions. GxE interactions can occur at the trait level with the best examples being studies that compared North American and New Zealand genetics under different feeding systems in New Zealand [18, 19] and Ireland [20–24]. Trait level GxE interactions among other countries are relatively modest [25]. The other main driver of GxE interactions is the differences in breeding goal, which can come through differences in economic values [26] and scale effects [27].

## Conclusions

Accounting for subpopulation substructure within a dairy industry is known to be important when evaluating opportunities to accelerate genetic progress. There is opportunity to increase economic benefits to dairy farmers by increasing domestic GS reliability when foreign genetic trends are lower than domestic genetic trends, for example, when large between-country G × E interactions exist. Conversely, if a foreign source of genetic material with a high rate of genetic progress is available, it will contribute substantially to the benefits of domestic genetic progress and the benefits of investments to improve the accuracy of GS locally will be substantially reduced.
